# Prey life‐history influences the evolution of egg mass and indirectly reproductive investment in a group of free‐living insect predators

**DOI:** 10.1002/ece3.8438

**Published:** 2021-12-27

**Authors:** Jean‐Louis Hemptinne, Emilie Lecompte, Arnaud Sentis, Anthony F. G. Dixon, Alexandra Magro

**Affiliations:** ^1^ Laboratoire Évolution & Diversité Biologique (UMR EDB 5174) Université de Toulouse CNRS IRD UPS Toulouse France; ^2^ INRAE Aix‐Marseille University UMR RECOVER Aix‐en‐Provence France; ^3^ School of Biological Sciences University of East Anglia Norwich UK; ^4^ Global Change Research Institute CAS Brno Czech Republic

**Keywords:** aphids, coccids, egg mass, insect predators, ladybird beetles, life‐history evolution, ovariole number, reproductive investment

## Abstract

1. The balance between risk and benefit of exploiting resources drives life‐history evolution in organisms. Predators are naturally recognized as major drivers of the life‐history evolution of their prey. Although prey may also influence the life‐history evolution of their predators in the context of an evolutionary arms race, there is far more evidence of the role of predators than of prey.
2. The goal of this study was to investigate the role of prey in life‐history evolution of predators using ladybird beetle predators of aphids and coccids. These particular ladybirds and their prey were chosen because literature shows that the pace of life of aphids is faster than that of coccids and this difference is reflected in the life histories of the ladybirds that specialize on feeding on aphids or coccids.
3. Thirty‐four species of ladybird predators of aphids and eight of coccids belonging to five different tribes were collected and reared in the laboratory. The females were weighed as well as their eggs, and their reproductive investment estimated as the number of ovarioles. Phylogenetic relatedness was controlled for in the statistical analyses.
4. Controlling for female mass revealed that ladybird predators of aphids lay bigger eggs than ladybird predators of coccids. This difference is not influenced by phylogenetic relatedness but only by the type of prey eaten. We suggest that ladybird predators of coccids lay smaller eggs because neonate larvae do not have to search, catch, and subdue prey. Both types of ladybirds have a similar reproductive investment relative to their body mass when phylogeny is controlled for.
5. Recognizing the influence of prey on the life‐history evolution of predators is important for understanding food web dynamics. From an applied perspective, this fine evolutionary tuning of prey–predator relationships should be used to guide and increase the efficiency of biological control programs.

## Graphical abstract

In insect predator, prey influences the evolution of life‐history traits. After controlling for phylogenetic relatedness, we show that ladybirds specialized on aphids lay bigger eggs than those that specialized on coccids. However, the two types of ladybirds invest equally in reproduction.

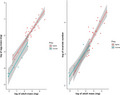

## 1 INTRODUCTION

In their quest for resources, organisms have to contend not only with competitors and natural enemies, but with resources that are not evenly distributed in space and time. This has resulted in an astounding diversity of life histories from generalists to specialists and slow to fast developers. In this context, the trade‐off between risks and rewards in exploiting specific resources drives the selection of life histories, which are characterized by a set of traits that optimally govern the allocation of energy to growth, survival, and reproduction (Bhat et al., 2020; Roff, 2002; Stearns, 1992). Contrary to Darwinian demons (Law, 1979) living in an imaginary world of unlimited resources where maximum simultaneous investment in all biological functions is possible, real organisms must optimally allocate limited resources between different functions (Burger et al., 2019; Roff, 2002). The fact that predators and prey may affect each other's life histories was suggested by Darwin (Abrams, 2001) and given further recognition when Cott (1940) coined the concept of an evolutionary arms race. However, although predator–prey coevolution generated a rich body of theoretical work (see Abrams, 2001 for a review; Scott et al., 2018), there is still far more evidence that free‐living predators act as selective forces for prey than vice versa (Abrams, 2001; Bhat et al., 2020). Reznick et al. (1990) provide an example of the evolutionary action of predators by demonstrating that the predatory fish *Crenicichla* select for lower age and size at maturity in guppies in streams in Trinidad. Conversely, Wilson et al. (2018) furnish a recent case of herbivores driving predator trait evolution in which impalas and zebras are adapted to counter the athletic performances of cheetah and lions, respectively.

In this paper, we study two life‐history traits of ladybird beetle predators.

There are 6000 species of ladybird beetles (Coleoptera, Coccinellidae) worldwide (Vandenberg, 2002). They evolved from mycophagous ancestors that switched to feeding on armored scales (Hemiptera, Coccidoidea) in the Early Cretaceous about 142.8 Mya ago at a time when Psylloidea and Coccidoidea already fed on primitive Angiosperms (Che et al., 2021; Giorgi et al., 2009; Magro et al., 2010; Seago et al., 2011; Song et al., 2019). They later underwent rapid speciation during the late Cretaceous, from 95 to 70 Mya. Seago et al. (2011) hypothesize that the trophic shift coupled with the acquisition of defensive dorsal glands, which protected the larvae of the common ladybirds’ ancestor from ants, further allowed ladybirds to diversify into a successful family predominantly specialized in feeding on soft‐bodied Sternorrhynchan insects (Hemiptera), such as, Aleyrodoidea, Psylloidea, Coccidoidea (coccids), and Aphidoidea (aphids) (Robertson et al., 2008), whereas Che et al. (2021) view the rapid speciation of ladybird beetles as an indirect evolutionary correlation of the quick diversification of Angiosperms in the late Cretaceous. It probably triggered the evolution of Sternorrhyncha insects, and more particularly aphids, which in turn enabled the rapid diversification of ladybirds.

Predation on Aphidoidea is therefore more recent than that on the sister group Coccidoidea (Johnson et al., 2018; Song et al., 2019) and independently evolved at least three times among ladybird beetles: firstly, twice in the tribe Coccidulini and, later, once in the tribe Coccinellini (Giorgi et al., 2009; Magro et al., 2010; Seago et al., 2011). Although the number of transitions from a coccid‐based regime to an aphid regime is low, we nevertheless decided to analyze the implication of these transitions on some life‐history traits because we are interested in understanding why coccidophagous ladybirds can regulate the abundance of their prey, whereas aphidophagous ladybirds do not (see the famous example of the Vedalia beetle in California (Dixon, 2000; Heimpel & Mills, 2017)). If specialization on a particular type of prey affects the evolution of predators’ life‐history traits, then Coccinellidae could be an ideal model group to study this question.

From an ecological point of view, the most striking difference between aphids and coccids is their developmental rate, with aphids developing 7 times faster than coccids (Dixon, 2000). Ladybirds that feed on aphids (*aphidophagous species*) also grow and move faster, have a higher metabolic rate, and tend to age more quickly than those feeding on coccids (*coccidophagous species*) (Dixon, 2000, 2015; Dixon & Honek, 2014; Dixon et al., 2016). These two groups of predators also differ in their reproductive behavior. The former lay their eggs some distance from aphid colonies. After hatching their larvae must hunt, catch, and subdue prey that are very mobile, exhibit several behavioral or chemical antipredator defenses (Dixon, 1958, 1998; Wu et al., 2010), and/or produce adaptive polyphenic morphs in response to the presence of predators (Dixon & Agarwala, 1999; Sentis et al., 2018). On the other hand, coccidophagous ladybirds lay their eggs in or on immobile ovisacs of their prey. Upon hatching the larvae start feeding on coccid eggs, which are rather small, inside these ovisacs (Dixon, 2000). That is, these two groups of predators have different life histories and consequently may also differ in the way they allocate their resources (Dixon & Hemptinne, 2001; Dixon et al., 1997).

Our first prediction states that the relative mass of the eggs of coccidophagous ladybirds should be lighter than those of aphidophagous ladybirds. It is based on the fact that unlike those of aphidophagous ladybirds the neonate larvae of coccidophagous species do not have to search for and subdue prey because they are born inside ovisacs of coccids: they feed on coccids eggs that surround them. Neonate larvae of aphidophagous ladybirds are born at some distance from aphid colonies. Therefore, they must find and then subdue aphids, which are not willing victims (see above). On average, neonate larvae of aphidophagous ladybirds have sufficient metabolic reserve at birth to hunt for two days without eating (Dixon, 1959).

The second prediction is that aphidophagous ladybirds should invest more resources in reproduction than coccidophagous ladybirds, which should invest more in energy reserves for fuelling foraging. Our second prediction is supported by field observations showing that, compared to coccids, the duration of aphid colonies is shorter, and they are less aggregated (Borges et al., 2011). This is because aphids thrive when plant sap is rich in nitrogen (Dixon, 1998; Douglas, 2003). They are therefore often extremely abundant in spring when most plants are growing, but much rarer in summer when plant growth is slower (Dixon, 1998; Karley et al., 2004). As female ladybirds only lay a single batch of eggs in each colony of aphids, a strategy that evolved to reduce intraspecific competition and cannibalism (Frechette et al., 2003; Hemptinne et al., 1992), their fitness depends on their ability to find enough colonies in which to lay eggs to take advantage of an abundant but very time‐limited resource. Coccids develop much slower than aphids, possibly because they do not feed on nitrogen‐rich phloem sap (Dixon et al., 2016). Thus, their colonies persist for longer than those of aphids (Borges et al., 2011). As these colonies are highly aggregated, however, it is likely that it takes their predators longer to locate them, as they spend more time searching for prey that is highly clumped (Ioannou et al., 2011; Taylor, 1977).

The aim of this paper is to test the robustness of these two predictions by respectively calculating the allometric relationships between egg mass and adult mass, and between reproductive investment and adult mass for forty‐two species of ladybirds that either feed on aphids or coccids. We incorporated phylogeny into our statistical analysis to control for shared ancestry and evaluate independent trait evolution using phylogenetic generalized least squares (PGLS) regressions (Symonds & Blomberg, 2014). We show that prey shape the evolution of egg mass, but not reproductive investment in this group of free‐living predators, which suggests that we should not underestimate the role of prey traits as a selective force for predators.

## 2 MATERIAL AND METHODS

### 2.1 Ladybirds

We obtained specimens of forty‐two species of ladybirds that belong to the subfamily Coccinellinae (thirty‐seven from the Palearctic Region, two from the Afrotropical Region, two from the Neartic Region, and one from the Neotropical Region; Table 1). Of the forty‐two species, thirty‐four are aphid predators and eight prey on coccids. The classification of ladybirds regarding their diet is binary. During reproduction in spring and summer, ladybird beetles are specialists: species that feed on coccids do not eat aphids, and vice versa. Coccid feeders are more specialized than aphid feeders because they only feed on a very limited number of species or even a single species. Most aphid feeders feed on several species of aphids although some species have a narrow prey range (Hodek, 1973; Hodek et al., 2012). A few of the specimens of each species was kept alive, brought back to the laboratory and reared while the rest was preserved in 95% ethanol for genetic analysis. In this paper, we follow the classification of Seago et al. (2011).

**TABLE 1:** List of the species, their prey, their origin, and the GenBank accession numbers of the sequences used in the phylogenetic analyses

Tribe and species	Prey	Origin	GenBank accession numbers		
			COI (651 pb)	18S (1862 pb)	28S (298 pb)
Tribe Chilocorini					
*Chilocorus bipustulatus* (L.)	Coccid	Toulouse (France)	HQ164771	GU073718*	GU073768*
*Exochomus quadripustulatus* (L.)	Coccid	UK (1)	GU073912*	GU073721*	GU073771*
Tribe Coccidulini					
*Coccinula quatuordecimpustulata* (L.)	Aphid	Toulouse (France)	GU073895*	GU073687*	GU073739*
*Cryptolaemus montrouzieri* Mulsant	Coccid	(2)	GU073908*	GU073708*	GU073758*
*Nephus bisignatus* Boheman	Coccid	Greece (3)	GU073909*	GU073709*	GU073759*
*Nephus includens* (Kirsch)	Coccid	Greece (3)	MN164642	GU073710*	GU073760*
*Nephus reunioni* Fürsch	Coccid	Cascais (Portugal)	MN164643	GU073711*	GU073761*
*Scymnus apetzi* Mulsant	Aphid	Algarve (Portugal)	GU073910*	GU073712*	GU073762*
*Scymnus interruptus* (Goeze)	Aphid	Algarve (Portugal)	GU073911*	GU073713*	GU073763*
*Scymnus nubilus* (Mulsant)	Aphid	Azores (Portugal; 4)	MW800601*	GU073714*	GU073764*
*Scymnus rubromaculatus* (Goeze)	Aphid	Greece	N.A.	GU073715*	GU073765*
*Scymnus subvillosus* (Goeze)	Aphid	Algarve (Portugal)	N.A.	GU073716*	GU073766*
*Rhyzobius lophantae* (Blaisdel)	Coccid	Algarve (Portugal)	N.A.	GU073725*	GU073775*
Tribe Coccinellini					
*Adalia bipunctata* (L.)	Aphid	Toulouse (France)	GU073889*	GU073675*	FJ621325
*Adalia decempunctata* (L.)	Aphid	Toulouse (France)	GU073888*	GU073674*	FJ621324
*Anatis ocellata* (L.)	Aphid	UK (1)	KX035143	GU073676*	GU073731*
*Calvia decemguttata* (L.)	Aphid	Gembloux (Belgium)	KX087252	MW781812*	N.A.
*Calvia muiri* (Timberlake)	Aphid	Fuchu (Japan; 1)	GU073890*	GU073678*	GU073733*
*Calvia quatuordecimguttata* (L.)	Aphid	UK (1)	HQ165298	GU073677*	GU073732*
*Cheilomenes lunata* (F.)	Aphid	Madagascar	GU073891*	GU073679*	GU073734*
*Cheilomenes sexmaculatus* (F.)	Aphid	Yamagata (Japan; 1)	KM244706	GU073680*	GU073735*
*Coccinella magnifica* Redtenbacher	Aphid	Ardennes (Belgium)	N.A.	GU073682*	GU073736*
*Coccinella miranda* Wallaston	Aphid	Canary Islands (Spain)	N.A.	GU073683*	GU073737*
*Coccinella quinquepunctata* L.	Aphid	UK (1)	N.A.	GU073684*	FJ621326
*Coccinella septempunctata* L.	Aphid	Toulouse (France)	GU073893*	AY748147	FJ621328
*Coccinella undecimpunctata* L.	Aphid	Lincoln (New Zealand)	GU073892*	GU073681*	FJ621327
*Coleomegilla maculata* (DeGeer)	Aphid	Québec (Canada; 5)	KP829555	GU073688*	GU073740*
*Eriopis connexa* Germar	Aphid	Chile (6)	MG253268	MW781813*	N.A.
*Harmonia axyridis* (Pallas)	Aphid	Kyoto (Japan; 7)	GU073896*	GU073689*	FJ621330
*Harmonia conformis* (Boisduval)	Aphid	Antibes (France; 8)	N.A.	GU073690*	GU073741*
*Harmonia dimidiata* F.	Aphid	(India; 9)	N.A.	MW781814*	N.A.
*Harmonia quadripunctata* (Pontoppidan)	Aphid	Toulouse (France)	GU073897*	GU073691*	FJ621329
*Hippodamia convergens* Guérin‐Méneville	Aphid	Texas (USA; 10)	KX755332	MW781815*	EU164644
*Hippodamia undecimnotata* Schneider	Aphid	Millau (France)	KX087298	GU073692*	GU073742*
*Hippodamia variegata* (Goeze)	Aphid	Algeria (11)	GU073898*	GU073693*	GU073743*
*Myzia oblongoguttata* (L.)	Aphid	Toulouse (France)	MF152813	GU073695*	GU073745*
*Oenopia conglobata* (L.)	Aphid	Toulouse (France)	N.A.	GU073697*	GU073747*
*Oenopia doublieri* (Mulsant)	Aphid	Algeria (11)	GU073900*	GU073696*	GU073746*
*Olla v*‐*nigrum* (Mulsant)	Aphid	Florida (USA; 12)	KP829565	GU073698*	GU073748*
*Propylea japonica* (Thunberg)	Aphid	Yamagata (Japan; 1)	HQ435808	GU073700*	GU073750*
*Propylea quatuordecimpunctata* (L.)	Aphid	Toulouse (France)	GU073901*	GU073699*	GU073749*
Tribe Noviini					
*Rodolia cardinalis (Mulsant)*	Coccid	Algarve (Portugal)	GU073916*	GU073726*	GU073776*

Note

Samples were collected by the authors with the following exceptions: 1: Drs R. Ware and M. Majerus (Cambridge, UK); 2: Purchased from Koppert; 3: Dr P. Milonas (Athens, Greece); 4: Dr I. Borges (Ponta Delgada, Portugal); 5: Dr B. Fréchette (Montréal, Canada); 6: Prof. A. Grez (Santiago, Chile); 7: Dr N. Osawa (Kyoto, Japan); 8: Dr E. Lombaert (Antibes, France); 9: Dr. O. Hemchandra (Imphā, Manipur, India); 10: Dr X. Martini (Quincy, FL, USA); 11: Dr L. Saharaoui (Alger, Algeria); 12: Dr J. A. Qureshi (North Immolakee, FL, USA).

For the DNA sequences: NA, sequence not available.*sequences acquired by the authors.

#### 2.1.1 Ladybird culture

Among the aphidophagous species, twenty‐eight belong to the tribe Coccinellini and six to the Coccidulini. In the laboratory, their sex was determined based on the shape of the last abdominal sternite (Hodek, 1973). Then, they were sorted into couples consisting of a female and a male that were each kept in a 9‐cm Petri dish, containing a piece of filter paper accordion folded to increase the surface area for oviposition, at 20 ± 1°C and a photo phase of 16 h for 2 weeks. Every day the ladybirds were transferred to a clean Petri dish and fed an excess of pea aphids, *Acyrthosiphon pisum* (Harris), which were reared on *Vicia faba* L. The eggs laid by the ladybirds on the folded filter paper were collected daily. The pea aphid is classified as “essential food” supporting normal reproduction for 10 of the species used in this study (Hodek et al., 2012). For the other species, it cannot be ruled out that this aphid is not the optimal prey. However, although prey quality affects clutch size, a study of Rana et al. (2002) suggests that it does not affect egg size in ladybirds.

Eight species of coccid feeding ladybirds (coccidophagous species) were either collected in the field or obtained from laboratory stock cultures. Two of them belong to the Tribe Chilocorini, five to the Coccidulini, and one to the Noviini. These species were sexed, paired, and reared as above but fed one of a greater diversity of prey because coccidophagous ladybirds are much more prey specific than aphidophagous species. *Cryptolaemus montrouzieri* Mulsant, *Nephus reunioni* Fürsch, *N. bisignatus* (Boheman), *N. includens* (Kirsch), and *Exochomus quadripustulatus* L. were fed *Planococcus citri* Risso, reared in darkness on potato sprouts. *Rodolia cardinalis* (Mulsant) was fed *Icerya purchasi* (Maskell) reared on *Pittosporum tobira* (Thunb.). *Rhizobius lophantae* (Blaisdell) and *Chilocorus bipustulatus* L. were fed *Aspidiotus nerii* Bouché, reared on potato tubers. As these ladybirds lay their eggs inside or below ovisacs of their prey, the ovisacs remaining in each Petri dish at the end of the day were dissected under a binocular microscope and searched for ladybird eggs.

#### 2.1.2 Mass of adults and eggs, and ovariole number

Female ladybirds were allowed to acclimatize to laboratory conditions for 10 days prior to weighing to an accuracy of 0.1 mg on a microbalance (Sartorius Supermicro S4 or SC2, Sartorius AG, Göttingen, Germany). The number of females weighed varied from 3 to 16 depending on the success we had in collecting and rearing each species (Table 1).

Eggs less than 24 h old were collected, separated from the substrate on which they were attached or inserted in, and weighed individually on the same microbalance to an accuracy of 0.1 mg. For each female, we intended to weigh 5 eggs from 5 successive ovipositions. However, some samples are smaller than 25 eggs because some field‐collected ladybirds did not survive long enough in the laboratory to oviposit five times.

After weighing, the females were humanely killed. Their elytra clipped off and abdominal tergites removed. Their ovaries were removed by seizing the oviduct with forceps and pulling them out of the abdominal cavity. They were placed on a microscope slide and stained with ethylene blue. Then, the numbers of ovarioles in both ovaries were counted under a binocular microscope and used to estimate the reproductive investment (definition given below).

#### 2.1.3 Ladybird phylogeny

Total genomic DNA was extracted from individual beetles after removing their elytra using the DNeasy Blood and tissue Kit from QIAGEN and following the PBS protocol according to the manufacturer's instructions. Two nuclear genes (18S rDNA and 28S rDNA) and one mitochondrial gene (COI) were amplified as described in Magro et al. (2010) (Table 1; but the 18S rDNA gene sequence was elongated in 5’ and 3’ using PCR primers 1F + a0.7 for 5’ end, and a2.0 + 9R for 3’ end; Whiting, 2002; Jarvis et al., 2004). Polymerase chain reactions were performed with 50 ng of DNA in 25 µl volumes containing a final concentration of 1X PCR buffer, 0.2 µM of each primer, 0.2 mM of each dNTPs, 1.5 mM of MgCl2, and 1 U of Taq polymerase. PCR settings for amplifying 18S fragments involved an initial denaturation of 4 min at 94°C, followed by 35 cycles of 60 s at 94°C, 1 min at 50°C, 60 s at 72°C and 10 min extension at 72°C. All PCR products were sequenced in both strands using Sanger sequencing technology. All raw reads were assembled using Geneious (v9.0.5; Biomatters, New Zealand) and manually checked for sequencing errors, ambiguities, and, if necessary, manually edited. The new sequences were deposited in GenBank under the accession numbers listed in Table 1.

The sequences were aligned using MAFFT (Katoh & Standley, 2013) for each gene separately, with default options, and the alignment was then reviewed and corrected by eye. The phylogenetic analyses were performed on the combined dataset with all three genes concatenated (2815 pb). In addition to the species reared in the laboratory, we also considered some species for which genetic information on the 18S rDNA and 28S rDNA and COI was available in GenBank (Table 1). The best‐fit model of evolution for the dataset was determined as the GTR+I+G using the Akaike information criterion (AIC), as implemented in SMS (Lefort et al., 2017).

Phylogenetic relationships were inferred based on maximum likelihood (ML) reconstruction and bootstrapping using RAxML 8.2.10 (Stamatakis, 2014) considering (1) no partition, (2) each gene as an independent partition (3 partitions), and (3) each codon position and each gene as an independent partition (5 partitions); individual alpha‐shape parameters, substitution rates, and base frequencies were estimated and optimized separately for each partition. Bootstrap support was determined using 100 pseudo‐replicates.

As the basal nodes in the RAxML trees were poorly supported alternative topologies were explored and phylogenetic relationships tested using other ML algorithms: (i) PhyML (Guindon & Gascuel, 2003) and 1000 bootstrap replicates to determine robustness of the nodes, and (ii) GARLI (Zwickl, 2006) implemented in Geneious (v9.0.5; Biomatters, New Zealand). The trees were rooted based on previous phylogenetic reconstructions of the family (Giorgi et al., 2009; Magro et al., 2010; Seago et al., 2011).

Phylogenetic relationships were also inferred using Bayesian inference in MrBayes v. 3.1.2 (Ronquist & Huelsenbeck, 2003). Two independent BI runs were carried out, each with four chains (with incremental heating) of 1,000,000 generations, with random starting trees, default priors (but with variable rates) and trees sampled every 1000 generations, using the GTR+I+G model estimated for all datasets. Stationarity was assessed graphically by plotting likelihood scores against chain generation and verifying that the standard deviation of split frequencies was under 0.01 (Ronquist & Huelsenbeck, 2003). For each run, the first 10,000 trees were discarded as burn‐in and the remaining trees used to construct a 50% majority‐rule consensus tree. The robustness of clades was assessed using clade posterior probabilities (PP).

In the end, we obtained six nonultrametric phylogenetic trees to control for relatedness in our analyses of the evolution of life‐history traits. The use of phylogeny with branch length unit (nonultrametric tree) that shows a phylogenetic signal improves the accuracy of comparative analyses (Litsios & Salamin, 2012).

### 2.2 Statistical analyses

The statistical analyses were performed in two steps.

First, we used a linear mixed effect (LMM) models with random intercepts and slopes to assess the relationships between log (egg mass) on one side, and log (adult mass) and food type (a factor with two levels: aphids and coccids) and their interaction on the other side. The models also included a random factor of individual ID number nested within ladybird species to account for the fact that several egg masses were recorded for each individual. We also included a random slope for ladybird species to account for potential interspecific variations in the slope of the relationship between log(egg mass) and log(adult mass).

For each individual, reproductive investment was the ovariole number. In a large analysis of insect taxa, Church et al. (2021) show that ovariole number is a reliable proxy for life‐time fecundity, which is an expression of reproductive investment. We use a generalized linear mixed‐effects model (GLMM) with a Poisson distribution and with random intercepts and slopes to assess the relationships between log (reproductive investment), and log (adult mass), food type, and their interaction. The structure of the random effects was similar as described above for the LMM model.

Linear mixed effects and GLMMs with random intercepts and slopes enabled the intraspecific variation in the relationships to be considered (Figure S1A,B). They were computed using the *lmer* function in the *lme4* package (R Core Team, 2020).

Then, the average values of egg masses, reproductive investments, and female masses were calculated for each species. The relationships between log (mean egg mass) and log (mean adult mass), and between log (mean reproductive investment) and log (mean adult mass) were analyzed using the phylogenetic generalized least square (PGLS) models in R’s packages ape and nlme (R Core Team, 2020; Symonds & Blomberg, 2014). The equations expressing egg mass or reproductive investment in relation to female body mass were expressed as gls functions with food type (aphid or coccid) as a covariate. These functions have a correlation argument that allows for quantify the strength of the phylogenetic signal; corPagel was used to calculate the value of Pagel's λ, which is the most used quantitative measure of a phylogenetic signal (Symonds & Blomberg, 2014). For each relationship, analyses of deviance were performed to test whether Pagel's λ was significantly different from 0 (no phylogenetic signal) or from 1 (strong phylogenetic signal). These analyses were run with each of the six phylogenetic trees that were computed (see above).

## 3 RESULTS

The average size of the females included in this study ranged from 0.9 to 77.6 mg. Once converted to body lengths using the ladybird mass–length relationship and data available in Hodek et al. (2012), it appears that the range and the distribution of the body sizes of the 42 species of ladybird beetles included in this study are representative of the Coccinellinae (Dixon & Hemptinne, 2001). On average, adult females belonging to the Coccinellini were twice as heavy as the Chilocorini and about five to ten times heavier than the Noviini and Coccidulini. The smallest eggs weighed 0.01 mg and the largest 0.68 mg. The variation in egg mass shows the same trends in the tribes as adult mass. The average value of the reproductive investment ranged from 7.8 to 90.0 ovarioles. The reproductive investment of Coccinelli was also the greatest but was only 2 or 3 times greater than that of Chilocorini, Coccidulini, or Noviini (Table 2).

**TABLE 2:** Average adult mass, egg mass, and reproductive investment per species in relation to the taxonomic position and prey consumed by the ladybirds in this study (*N* stands for the number of species, SD for standard deviation)

Tribe	Prey	*N*	Adult mass (mg)		Egg mass (mg)		Reproductive investment (ovariole number)	
			Mean	SD	Mean	SD	Mean	SD
Chilocorini	Coccid	2	10.509	1.581	0.123	0.013	24.21	2.33
Coccidulini	Aphid	6	2.073	0.659	0.032	0.012	11.76	1.26
Coccidulini	Coccid	5	3.554	4.686	0.025	0.024	13.70	6.09
Coccinellini	Aphid	28	29.975	22.122	0.226	0.123	39.98	17.35
Noviini	Coccid	1	5.489		0.034		27.10	

**FIGURE 1:**
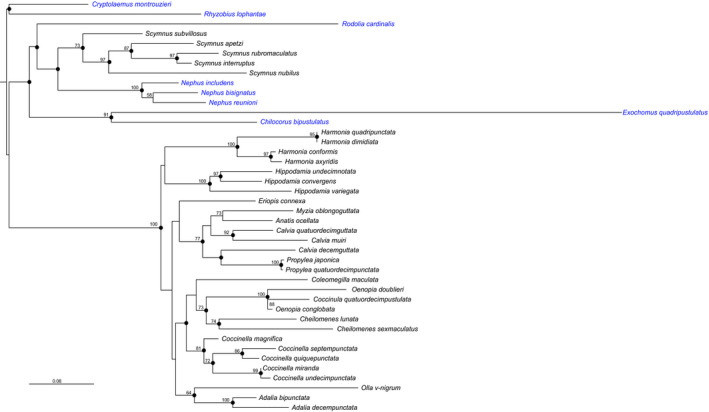
Phylogenetic tree of the 42 species of Coccinellinae included in this study. The topology and branch lengths are from the RAxML analysis conducted with three partitions (1 per gene). Nodes congruent between all reconstruction methods are indicated by black circles; numbers beside nodes are bootstrap values. The coccidophagous species are in blue and the aphidophagous in black

The phylogenetic analyses provided slightly different topologies for the six trees depending on the algorithm and whether or not a partitioned strategy was used (RAxML). However, most of the nodes were consistently recovered in the different analyses: the monophyly of the tribes Chilocorini and Coccinellini and several clades within Coccidulini and Coccinellini were congruent between all reconstructions and often well supported (Figure 1). However, the higher level relationships among the Coccinellidae and the Coccinellini tribe were poorly supported (Figure 1). The two main differences between the trees were the relationships between the genera *Harmonia* and *Hippodamia*, which are either sister groups when a partition strategy was used, or not so in trees without partition, in which *Harmonia* is the first clade to diverge, and the relative positions of some species, such as *Eriopis connexa* and *Coleomegila maculata*, within the Coccinellini clades.

### 3.1 Adult and egg masses

**FIGURE 2:**
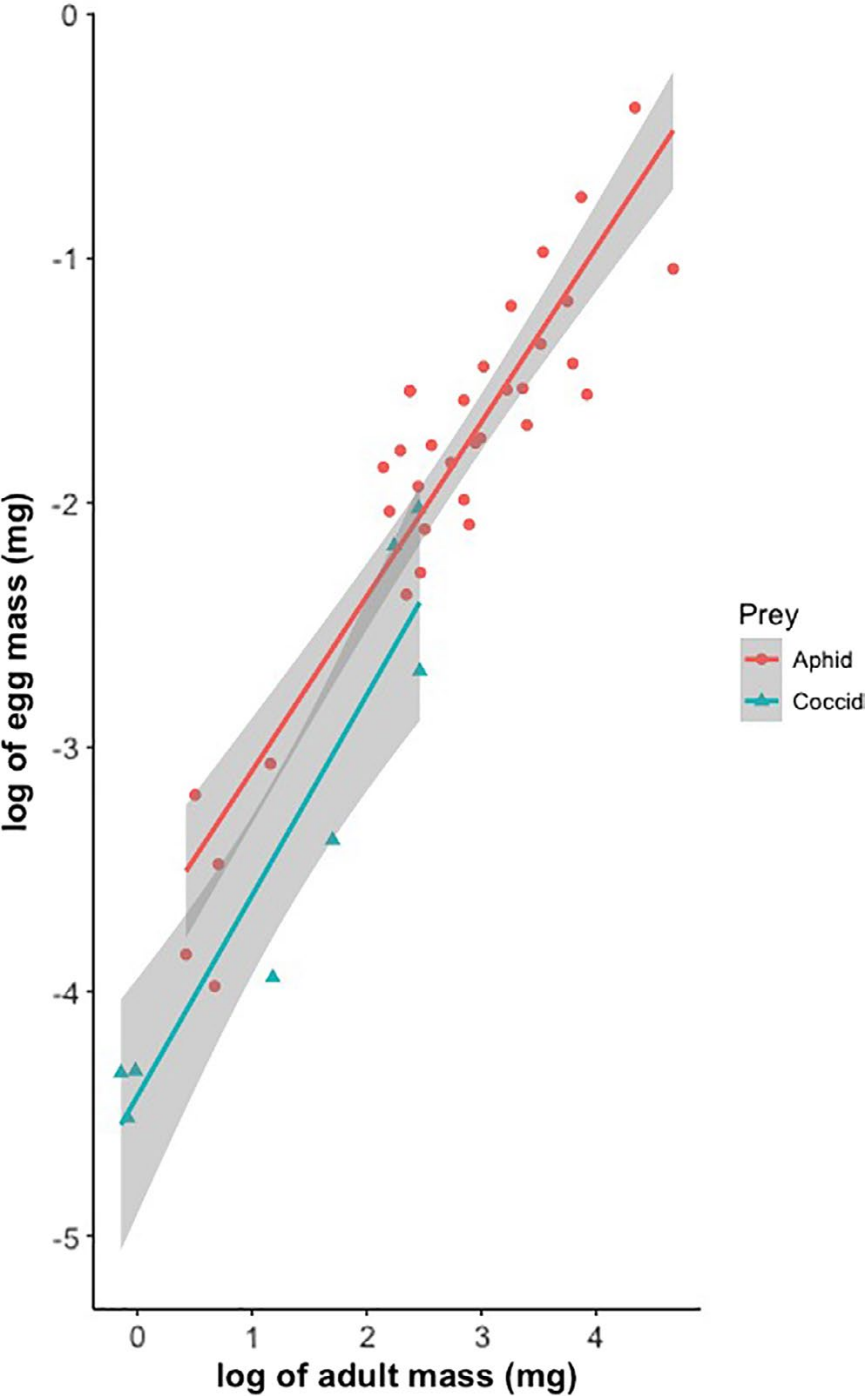
The relationship between log egg mass and log adult mass for 42 species of aphidophagous and coccidophagous ladybird beetles

As in the LMM, the interaction between log (adult mass) and food type was not significant (*p* > .05). It was not included in the final model. Log (egg mass) is not associated with log (adult mass) indicating that large females do not produce relatively larger eggs. However, food type has a significant effect because coccidophagous ladybirds lay significantly smaller eggs than aphidophagous species (Figure 2; Table 3).

**TABLE 3:** Summary of the linear mixed models (LMM) of the effect of adult mass and food type (aphid or coccid) on egg mass and of the generalized linear mixed model (GLMM) of the effect of adult mass and food type (aphid or coccid) on the reproductive investment of 42 species of ladybird beetles

	Log (egg mass)			Log (reproductive investment)		
	Estimate (SD)	*t* value	*p* value	Estimate (SD)	*z* value	*p* value
Model with interaction						
Log (adult mass)	0.0357 (0.0524)	0.682	.498	0.0035 (0.0019)	1.828	.0676
Food type: Coccid	−1.5569 (0.3469)	−4.488	5.65 × 10^−5^	−0.6568 (0.2241)	−2.931	.0034
Interaction	0.0633 (0.1097)	0.577	.567	0.0143 (0.0200)	0.716	.4741
Model without interaction						
Log (adult mass)	0.0508 (0.0459)	1.406	.274	0.0035 (0.0019)	1.832	.0670
Food type: Coccid	−1.4889 (0.3257)	−4.571	6.50 × 10^−5^	−0.5772 (0.1975)	−2.922	.0035

For the PGLS analyses, the interactions between adult mass and food type were never significant whatever phylogenetic tree was used (*p* > .05) and therefore also omitted. The models without interaction confirmed the results of the LMM analysis in indicating that log (egg mass) significantly scales with log (adult mass) with an exponent ranging from 0.646 to 0.737 depending on the phylogenetic tree used in the analysis (Table 4). Coccidophagous ladybirds lay significantly smaller eggs than aphidophagous species. Although the value of Pagel's λ coefficient is influenced by the nature of the phylogenetic tree included in the PGLS analysis, the analyses of deviance indicate that the λ values are always significantly different from 1 but not from 0 (no phylogenetic signal) (Table 5). This indicates that the difference in egg mass is associated with the kind of prey hunted by the ladybirds rather than a consequence of phylogenetic relatedness.

**TABLE 4:** Summary of the PGLS models without the interaction term (log(mass mean)*food type) used to analyze the effect of adult mass and food type (aphid or coccid) on egg mass and reproductive investment of 42 species of ladybird beetles for each of the 6 phylogenetic trees assembled in this study

Phylogenetic tree	Log (egg mass)			Log (reproductive investment)		
	Estimate (SD)	*t* value	*p* value	Estimate (SD)	*t* value	*p* value
Maximum likelihood						
No partition^a^						
Intercept	−3.895 (0.173)	−22.460	.000	2.180 (0.158)	13.763	.000
Log(mass mean)	0.719 (0.050)	14.486	.000	0.452 (0.051)	8.937	.000
Food type (coccid)	−0.488 (0.143)	−3.4001	.002	0.060 (0.148)	0.404	.688
3 partitions^a^						
Intercept	−3.822 (0.135)	−28.353	.000	2.178 (0.102)	21.236	.000
Log(mass mean)	0.730 (0.048)	15.277	.000	0.468 (0.035)	13.120	.000
Food type (coccid)	−0.510 (0.152)	−3.363	.002	0.410 (0.109)	0.376	.709
5 partitions^a^						
Intercept	−3.895 (0.173)	−22.460	.000	2.194 (0.105)	20.836	.000
Log(mass mean)	0.719 (0.050)	14.486	.000	0.464 (0.036)	12.780	.000
Food type (coccid)	−0.488 (0.143)	−3.401	.002	0.052 (0.112)	0.426	.646
PhyML^b^						
Intercept	−3.895 (0.173)	−22.460	.000	2.205 (0.101)	21.764	.000
Log(mass mean)	0.719 (0.050)	14.486	.000	0.455 (0.034)	13.362	.000
Food type (coccid)	−0.488 (0.143)	−3.401	.002	0.038 (0.107)	0.356	.724
GARLI^c^						
Intercept	−3.924 (0.153)	−25.578	.000	2.154 (0.097)	22.177	.000
Log(mass mean)	0.728 (0.049)	14.701	.000	0.457 (0.035)	12.939	.000
Food type (coccid)	−0.466 (0.145)	−3.205	.003	0.070 (0.108)	0.648	.521
Bayesian inference^d^						
Intercept	−3.891 (0.136)	−28.522	.000	−1.763 (0.108)	−16.381	.000
Log(mass mean)	0.737 (0.046)	15.836	.000	1.223 (0.040)	30.795	.000
Food type (coccid)	−0.421 (0.144)	−2.927	.006	−0.308 (0.130)	−2.378	.022

^a^ Maximum likelihood reconstruction and bootstrapping with RAxML v 8.2.10 with successively no partition, 3 partitions (each gene as an independent partition), and 5 partitions (each codon and each gene as independent partitions).

^b^ Maximum likelihood reconstruction and bootstrapping with PhyML.

^c^ Maximum likelihood reconstruction and bootstrapping with GARLI in Geneious v 9.0.5.

^d^ Bayesian inference with MrBayes v 3.1.2.

**TABLE 5:** The minimum and maximum values of Pagel's λ calculated using the PGLS models without the interaction term (log(mass mean)*food type), which were used to analyze the effect of adult mass and food type (aphid or coccid) on egg mass and reproductive investment of 42 species of ladybird beetles for each of the 6 phylogenetic trees assembled in this study. Deviance analyses were performed to assess whether the values of λ differed from 0 (no phylogenetic signal) and 1 (strong phylogenetic signal)

Phylogenetic tree	Log (egg mass)			Log (reproductive investment)		
	Pagel's λ (min – max)	Analysis of deviance (*p* value)		Pagel's λ (min – max)	Analysis of deviance (*p* value)	
		λ = 0	λ = 1		λ = 0	λ = 1
Maximum likelihood						
No partition^a^	0.611 (0.219 to 1.003)	.041	.000	0.478 (−0.636 to 1.019)	.195	.0001
3 partitions^a^	−0.059 (−0.063 to −0.056)	.347	.000	−0.086 (−0.105 to 0.067)	.259	.0001
5 partitions^a^	0.521 (−0.142 to 1.185)	.347	.000	−0.043 (−0.264 to 0.179)	.764	.0001
PhyML^b^	0.521 (−0.142 to −1.185)	0.347	.000	−0.068 (−0.070 to −0.066)	.122	.001
GARLI^c^	0.210 (−0.524 to 0.944)	.579	.000	−0.228 (−0.252 to −0.204)	.114	.0001
Bayesian inference^d^	−0.075 (−0.084 to −0.065)	.243	.000	−0.086 (−0.105 to −0.067)	.259	.0001

^a^ Maximum likelihood reconstruction and bootstrapping with RAxML v 8.2.10 with successively no partition, 3 partitions (each gene as an independent partition), and 5 partitions (each codon and each gene as independent partitions).

^b^ Maximum likelihood reconstruction and bootstrapping with PhyML.

^c^ Maximum likelihood reconstruction and bootstrapping with GARLI in Geneious v 9.0.5.

^d^ Bayesian inference with MrBayes v 3.1.2.

### 3.2 Reproductive investment and adult masses

**FIGURE 3:**
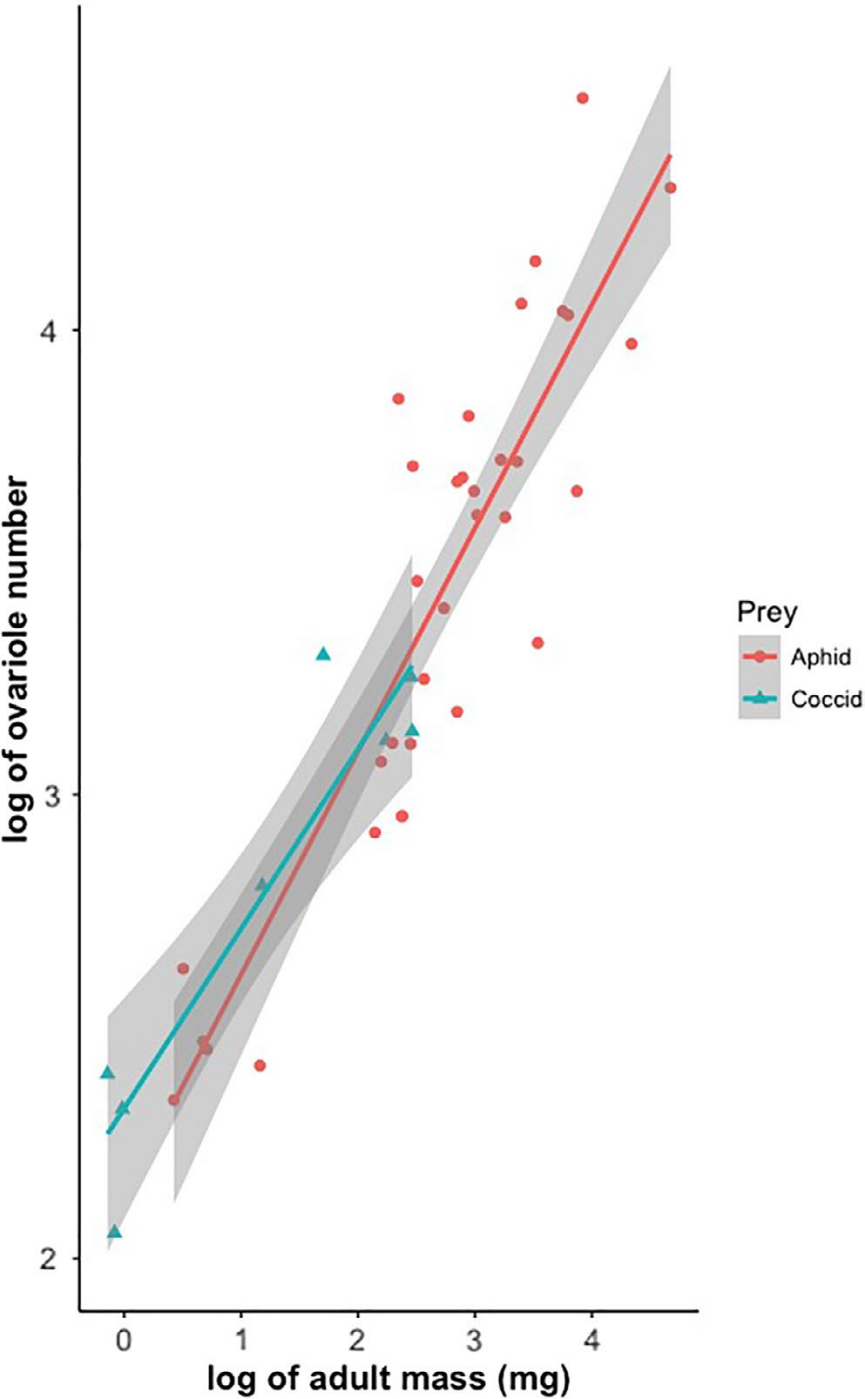
The relationship between log reproductive investment and log adult mass for 42 species of aphidophagous and coccidophagous ladybird beetles

For the GLMM, the interaction between log (adult mass) and food type was not significant (*p* > .05) (Table 3), so it was not included in the final model. Log (reproductive investment) is marginally associated with log (adult mass), with coccidophagous ladybirds having significantly smaller reproductive investment than those feeding on aphids (Table 3). However, the slopes between log(adult mass) and log(ovariole number) in the two groups of ladybirds are not different from each other (Figure 3).

For the PGLS analyses, the interactions between adult mass and food type were also not significant (*p* > .05) and therefore not included in the analyses (Table 3). The models without interactions show that log (reproductive investment) significantly scales with log (adult mass) with an exponent ranging from 0.452 to 0.465 depending on the method used to construct the phylogenetic tree. However, the reproductive investment of coccidophagous ladybirds is not smaller than that of aphidophagous species (Table 4).

## 4 DISCUSSION

In this paper, we investigated the evolution of egg mass and reproductive investment of two groups of ladybird beetle predators to look at the role of prey as a selective force for the evolution of the life history of predators taking phylogeny into account to control for shared ancestry. The PGLS analyses validated our first prediction: eggs of aphidophagous ladybirds are relatively heavier than those of coccidophagous species. However, our second prediction is not supported by the PGLS analyses: coccidophagous ladybirds do not invest less in reproduction than aphidophagous ones.

The range and the distribution of the body sizes of the 42 species of ladybird beetles included in this study is representative of the Coccinellinae (Dixon & Hemptinne, 2001). Eggs are rarely weighed, and the numbers of ovarioles barely quoted in the literature. Therefore, our measures constitute an original dataset of specific egg masses and ovariole numbers. Once accounting for phylogeny, these data reveal that egg masses are always significantly and positively related to the mass of the females with exponents like those previously obtained for 8 species of aphidophagous ladybirds by Stewart et al. (1991). In addition and depending on the phylogenetic tree used in the analysis, the values of this exponent are between 0.65 and 0.74 and are therefore close to the 0.75 predicted by the Metabolic Theory of Ecology for processes of biomass production (Brown et al., 2004). The correlation for reproductive investment is not significant.

Ladybird beetles evolved from mycophagous ancestors by firstly becoming predators of coccids some of which evolved into predators of other soft‐bodied Sternorrhyncha (Hemiptera), such as psyllids or aphids during a fast radiative speciation process (Giorgi et al., 2009; Magro et al., 2010; Seago et al., 2011). It occurred during the late Cretaceous, from 120 to 70 Mya, at the same time as the fast diversification of Angiosperms and the appearance and rapid diversification of aphids (Che et al., 2021). Our study focused on coccid and aphid feeders distributed in different lineages, according to the revised classification of Seago et al. (2011). It shows that in ladybirds the type of prey and not phylogeny is most likely to have determined the evolution of the egg mass. We excluded other causes of transitions from our analysis because there is no indication that the shift from a coccid to an aphid regime could have coincided with a geographic shift (Dixon et al., 1987). On the contrary, aphids and coccids live in the tropics and temperate regions where they occupy different niches on plants. Interactions with ants are also good candidates to explain the evolution of feeding regimes; however, the information is scanty, and the most recent review of the subject indicates it occurs in coccidophagous as well as aphidophagous species (Majerus et al., 2007).

Our first hypothesis was that coccidophagous ladybirds should lay smaller eggs than aphidophagous species because larvae do not need to search for and subdue prey unlike aphidophagous larvae. We confirm this hypothesis. Our second hypothesis stated that coccidophagous species should have smaller reproductive investment, which was estimated by ovariole numbers (Church et al., 2021), compared to aphidophagous ladybirds because they have to allocate more resources to searching for prey. Contrary to aphids, coccids are more difficult to find because they are highly aggregated (Ioannou et al., 2011; Taylor, 1977). The GLMM indicates that coccidophagous species have fewer ovarioles than aphidophagous species. It also shows that ovariole numbers are marginally associated with adult body mass, and the coccidophagous species in our sample tend to be smaller than aphidophagous species. However, when taking phylogeny into account, it appears that ovariole number is only significantly related to adult body mass and not to the type of prey. Therefore, we cannot confirm the second hypothesis.

For the purpose of our study, the relative positions of the tribes Noviini and Chilocorini, which contain coccidophagous species, of the tribe Coccidulini, with both coccidophagous and aphidophagous species, and of the tribe Coccinellini, which hosts aphidophagous species, are of crucial importance to evaluate the role of phylogeny in the evolution of egg mass and reproductive investment. Our phylogenetic reconstructions are similar to previous studies; they have the same limitations due to incomplete resolutions and show analogous relationships between taxa (Che et al., 2021; Giorgi et al., 2009; Magro et al., 2010; Seago et al., 2011). Despite weak support, the relative positions of Noviini, Chilocorini, Coccidulini, and Coccinellini are congruent between the different reconstruction methods but also with former analyses (Che et al., 2021; Magro et al., 2010). We also recovered the monophyly of Chilocorini and Coccinellini, as well as of several clades within Coccinellini (Escalona et al., 2017; Giorgi et al., 2009; Magro et al., 2010), bearing in mind that all these studies are not based on the same set of species. In the recent study of Che et al. (2021), a larger sample of species combined with a deeper gene coverage did not resolve the relationships between all the tribes of the Coccinellidae. Therefore, the phylogeny of the ladybirds probably reflects the rapid diversification of these beetles during the Cretaceous (Che et al., 2021).

In all the phylogenetic studies on Coccinellidae, the basal relationships subtend extremely short branches like polytomy (Che et al., 2021; Giorgi et al., 2009; Magro et al., 2010; Robertson et al., 2015; Seago et al., 2011). This could influence the quantification of the phylogenetic signal because Pagel's λ is based on the Brownian motion model of trait evolution (Pagel, 1999), where trait evolution follows a random walk along the branches of the phylogenetic tree. The variance in the distribution of trait values is directly proportional to branch length. Yet, it has been shown that Pagel's λ is robust for incompletely resolved phylogenies, including polytomies, or branch length information (Molina‐Venegas & Rodríguez, 2017; Münkemüller et al., 2012). Thus, because our statistical results are consistent in the face of our different phylogenetic reconstructions, we are confident that our conclusions regarding the absence of a phylogenetic signal in the evolution of egg mass in ladybird beetle predators.

Although most studies emphasize the effect of predators on the evolution of the life‐history traits of their prey (review in Abrams, 2001), our study differs in documenting the role of prey in the evolution of egg mass of predators. It is possible that the paucity of such studies may simply be that mortality inflicted by predators is such an obvious penalty in terms of prey fitness that it stimulated more research interest. Another explanation is that the food consumed by predators is not perceived as limiting because it is rich and well‐balanced in energy and nitrogen (Ugine et al., 2018), whereas plant tissues are poorer in nitrogen, rich in fibers, and protected by arrays of defensive secondary chemicals. Therefore, herbivores are engaged in arms races with their food sources that have resulted in a great diversity of life histories (Agrawal, 2007).

However, the high nutritional quality of the food of predators should not obscure the fact that prey is far from being easily accessible. Contrary to expectations, however, a high proportion of prey is defended by toxins taken up from their food and sequestered in their tissues (Erb & Robert, 2016; Glendinning, 2007) and others are protected by ants (Majerus et al., 2007; Sentis et al., 2012; Vantaux et al., 2012). For example, *Chrysopa slossonae* Banks (Neuroptera) that feeds only on ant‐tended woolly alder aphids *Prociphilus tesselatus* (Fitch) is larger, less fecund and produces larger eggs than its sister species, *C. quadripunctata* Burmeister, which is a generalist predator of aphids (Albuquerque et al., 1997). Prey distribution in space and time constitute another important risk for predators and is a strong driver of life‐history evolution (Bhat et al., 2020; Kramer, 2001).

The association of our first prediction with ecological circumstances relevant to ladybirds is straightforward. Coccidophagous species tend to lay a single or very few eggs in or on ovisacs of their prey, which is a hard shell or a ball of white waxy filaments. (Hodek et al., 2012). On hatching, the neonate larvae do not have to forage for or subdue their prey but simply eat the coccid eggs that surround them. If the action of an oviposition‐deterring pheromone reported by Merlin et al. (1996) in *Cryptolaemus montrouzieri* also regulates the oviposition behavior in other species, then the young larvae would experience a low level of intraspecific competition. In contrast, aphidophagous species lay batches of eggs some distance from aphid colonies, with aphids being very mobile and able to defend themselves in various ways (Dixon, 1998; Hartbauer, 2010). The size at birth of larvae of aphidophagous species is therefore critical because neonate larvae must have enough energy to search, locate, catch, subdue, and eat their first prey (Dixon, 1958, 1959). That large neonate larvae can survive for longer searching for their first meal (Hodek et al., 2012) is likely to have resulted in aphidophagous ladybirds being selected to lay bigger eggs than coccidophagous species. We think this is the most parsimonious explanation because it concerns the survival of eggs, which are the most vulnerable stage of development in insects (Hironori & Katsuhiro, 1997; Ponsonby & Copland, 1998). Myrmecophily could also act on the evolution of egg size. However, ants tend coccids as well as aphid colonies (Majerus et al., 2007).

We formulated the second prediction (a lower reproductive investment in coccidophagous ladybirds) based on the results of two studies on the allocation of fat to gonads and somatic tissues in similar‐sized aphidophagous and coccidophagous ladybirds (Borges et al., 2011; Magro et al., 2003). Fat is important for both oogenesis and as a source of energy in insects (Bursell, 1970; Chapman, 1998; Herz & Heitland, 2002; Wheeler, 1996). The percentage of total body fat in the gonads of aphidophagous *Adalia bipunctata* (L.) is 37% compared to only 27% in the case of the similar‐sized coccidophagous *Cryptolaemus montrouzieri* Mulsant (Magro et al., 2003). The difference is even greater for two Coccidulini ladybirds living in similar habitats with 16% reported for the aphidophagous *Scymnus nubilus* Mulsant and only 5.5% for the coccidophagous *Nephus reunioni* Fürsch (Borges et al., 2011).

That there is a higher percentage of the total fat content in the soma of coccidophagous than aphidophagous ladybirds means it is likely that they have more fuel for dispersal, which may reflect their need to spend more time searching for oviposition sites than aphidophagous species. This is supported by the distribution of aphid and coccid colonies in natural vegetation with aphids occurring randomly mainly in rather ephemeral large colonies in habitats where coccids form smaller but longer lasting and highly aggregated colonies (Borges et al., 2011). Because of their clumped distribution it takes longer for natural enemies to find such prey (Ioannou et al., 2011; Taylor, 1977). Therefore, it is likely that selection favored coccid predators that allocated a greater proportion of their resources to fat for foraging than aphidophagous species. However, our analyses do not fully support this prediction; ovariole number is related to female body mass but not to the type of prey eaten when phylogeny is considered. This result could stem from the choice of the proxy for reproductive investment. There are two kinds of proxies: those related to ovarian development and one using the number of ovarioles to assess lifetime fecundity (Church et al., 2021; Cini et al., 2013). We can estimate ovarian development by multiplying the number of ovarioles by the mass of an egg, which is close to that of mature oocytes. However, as a prediction based on this proxy is not independent of our first prediction on egg mass, we choose the second type of proxy based on ovariole number (Church et al., 2021). If coccidophagous and aphidophagous ladybirds have the same reproductive investment relative to their body mass, but the former live slower and have a longer reproductive period than the later (Borges et al., 2011; Dixon, 2015), then the reproductive investment of coccidophagous ladybirds per day of life should be lower than that of aphidophagous ladybirds.

This study would probably benefit from a larger sample of Coccinellinae species that better reflect the diversity of trophic specializations in ladybirds. However, we were limited in our capacity for rearing ladybirds for collecting eggs by the high food specificity of most coccid feeding ladybirds and some aphidophagous species, and by the daunting task of maintaining many specific cultures of prey for rearing the ladybirds. In addition, correlative studies such as this would benefit greatly if it could examine more than 2 or 3 transitions from coccidophagy to aphidophagy in the Coccinellidae, but we cannot overcome this limitation because of the adaptive radiation in ladybirds (Che et al., 2021; Seago et al., 2011). A possibility of overcoming this limitation would be to show that the results are general to natural enemies and not just specific for ladybirds. The hoverflies (Diptera, Syrphidae), lacewings (Neuroptera), and hymenopterous parasitoids are also natural enemies of soft‐bodied Sternorrhynchan insects (Hemiptera) (Canard, 2001; Dziock, 2005; Miller et al., 2004) and deserve more attention in this respect, but unfortunately their life histories are still less well known. However, an analysis of the rate of development of 17 species of hymenopterous parasitoids shows that those parasitizing aphids develop twice as fast as those attacking coccids (Dixon & Honek, 2014). It gives support to our study because it already suggests that prey traits may also shape life histories of natural enemies other than ladybirds.

Although the two comparative studies on the allocation of resources to the gonads and soma of similar‐sized species of ladybirds lend some support to our results (Borges et al., 2011; Magro et al., 2003), a broader picture of the relationship between reproductive investment and other traits is missing. The simple fact that longevity under natural conditions is unknown for ladybirds precludes a clear vision of the contribution of particular traits and the trade‐offs among them, to demography and fitness (Laughlin et al., 2020). Estimating longevity still remains a challenge because ladybirds are rather small, mobile in their breeding habitats, and migrate to hibernation sites that are not always known (Hodek et al., 2012).

Food quality and temperature are among the drivers of the evolution of life‐history traits of herbivorous insects (Clissold & Simpson, 2015). Therefore, one cannot exclude that they may also explain the evolution of the reproductive investment of ladybird predators through their influence on coccid and aphid life history. In terms of food quality, coccids may require a longer handling time than aphids because of the nature of their cuticle or because they may sequester more defensive secondary compounds from plant sap. However, we did not find support for that in the literature. In addition, coccidophagous ladybirds mainly consume coccid eggs, which are likely supplied with the correct nutrient balance for embryonic development (Dixon et al., 2011). Regarding temperature, many species from the major subfamilies of aphids are endemic to the tropics and subtropics and thrive in the climatic conditions that prevail there. Like temperate species, tropical aphids differ from coccids by their fast pace of life that combines telescoping generations and parthenogenesis (Dixon et al., 1987). As our sample of ladybirds contain aphidophagous and coccidophagous species from both tropical and temperate countries, we believe temperature does not appear to be the most likely factor shaping ladybird life histories.

In conclusion, we have shown that when the confounding effect of phylogeny is removed, it is likely that the specialization of ladybirds on aphids and coccids resulted in them laying eggs of different sizes. As coccidophagous ladybirds have a slower pace of life than aphidophagous species, it is also possible that their reproductive investment per day is lower than that of aphidophagous ladybirds. Because of the much faster pace of life of aphids compared to coccids, it is likely that the traits of coccidophagous ladybirds are closer to those of the ancestral form from which aphidophagous ladybirds evolved. This fine evolutionary tuning of prey–predator relationships should guide biological control programs as is well illustrated by the particular case of aphid and coccid pests (Kindlmann & Dixon, 1999; Kindlmann et al., 2021; Mills, 2018).

## CONFLICT OF INTEREST

None of the authors of this manuscript has declared any conflict of interest.

## AUTHOR CONTRIBUTIONS

**Jean‐Louis Hemptinne:** Conceptualization (equal); Data curation (equal); Formal analysis (equal); Funding acquisition (equal); Investigation (equal); Methodology (equal); Project administration (equal); Resources (equal); Software (equal); Supervision (equal); Validation (equal); Visualization (equal); Writing – original draft (equal). **Emilie Lecompte:** Data curation (equal); Formal analysis (equal); Methodology (equal); Writing – review & editing (equal). **Arnaud Sentis:** Formal analysis (equal); Writing – review & editing (equal). **Anthony F. G. Dixon:** Conceptualization (equal); Methodology (equal); Writing – review & editing (equal). **Alexandra Magro:** Data curation (equal); Funding acquisition (equal); Investigation (equal); Project administration (equal); Resources (equal); Supervision (equal); Writing – review & editing (equal).

## Supporting information

Figure S1

## Data Availability

All the gene sequences are available in GenBank. Data on life‐history traits is available at https://doi.org/10.5061/dryad.pg4f4qrqz.
