# Supplementary figures and images for: Prey life‐history influences the evolution of egg mass and indirectly reproductive investment in a group of free‐living insect predators
Source: Ecol Evol. 2021 Dec 27;12(1):e8438. doi: 10.1002/ece3.8438 (PMC8796932; doi:10.1002/ece3.8438)

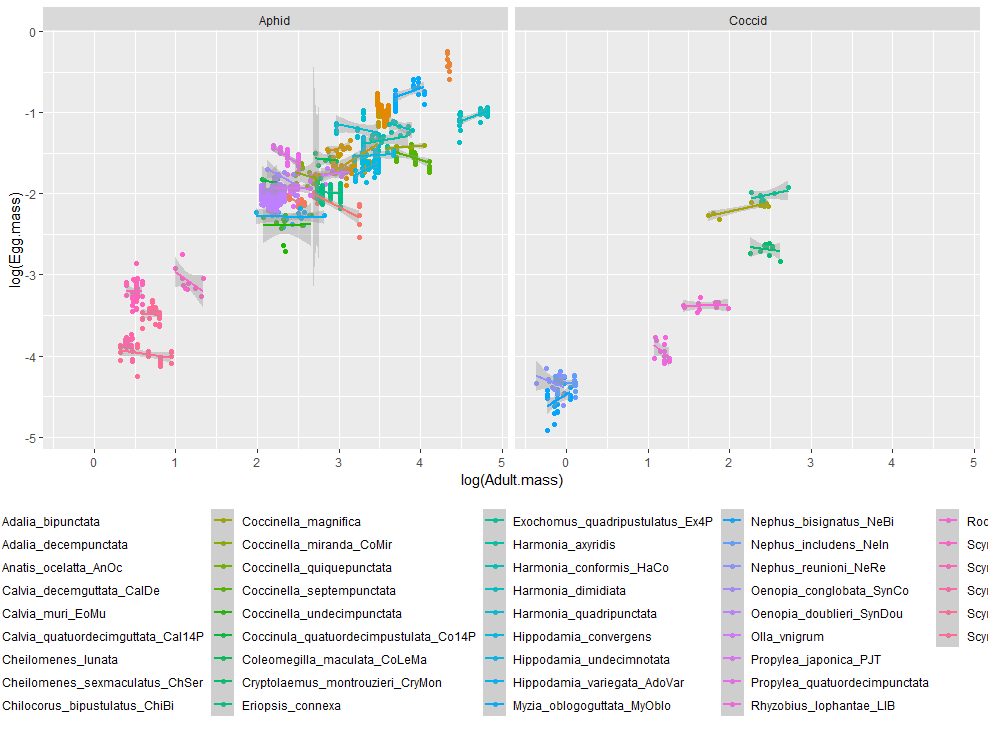

Supplement: Supplementary file 1 — Figure S1 [file ECE3-12-e8438-s001.zip › ece38438-sup-0001-FigS1A.tiff]

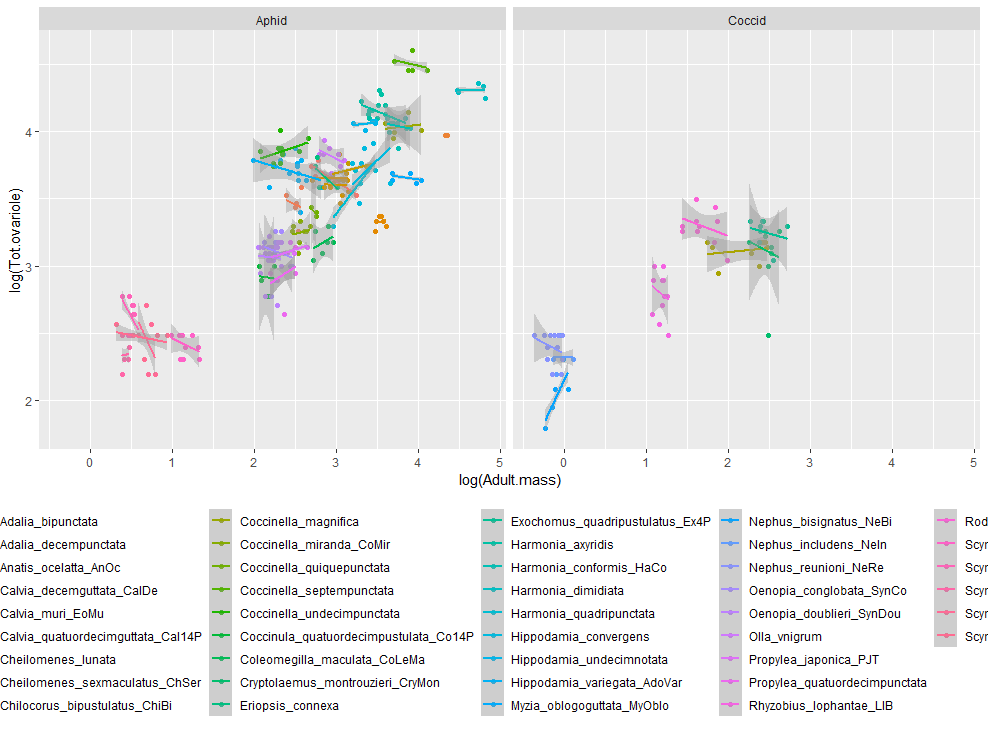

Supplement: Supplementary file 1 — Figure S1 [file ECE3-12-e8438-s001.zip › ece38438-sup-0002-FigS1B.tiff]
